# Evaluation of HER2 immunohistochemistry expression in non-standard solid tumors from a Single-Institution Prospective Cohort

**DOI:** 10.37349/etat.2024.00265

**Published:** 2024-08-22

**Authors:** Saurav Verma, Amanda Chapman, Lee-Anne Pickard, Danielle Porplycia, Haley McConkey, Patricia Jarosz, James Sinfield, Carolyn Lauzon-Young, Matthew J Cecchini, Christopher Howlett, Natalie Grindrod, Bekim Sadikovic, Stephen A Welch, Daniel Breadner

**Affiliations:** IRCCS Istituto Romagnolo per lo Studio dei Tumori (IRST) “Dino Amadori”, Italy; ^1^Division of Medical Oncology, Department of Oncology, Schulich School of Medicine & Dentistry, Western University, London, ON N6A 5C1, Canada; ^2^Verspeeten Family Cancer Centre, London Health Sciences Centre, London, ON N6A 5W9, Canada; ^3^Department of Medicine, Schulich School of Medicine & Dentistry, Western University, London, ON N6A 5C1, Canada; ^4^Department of Pathology and Laboratory Medicine, London Health Sciences Centre, London, ON N6A 5W9, Canada; ^5^Department of Pathology and Laboratory Medicine, Schulich School of Medicine & Dentistry, Western University, London, ON N6A 5C1, Canada

**Keywords:** HER2, IHC, solid tumors, biomarker

## Abstract

**Aim:**

Human epidermal growth factor receptor-2 (HER2) is a well-established prognostic and predictive biomarker. It is an FDA-approved therapeutic target for HER2 positive breast, gastroesophageal, and more recently, lung and colon cancers. It is an emerging biomarker in biliary tract, bladder, cervical, endometrial, ovarian, and pancreatic cancers. The emergence of new indications warrants further characterization of HER2 expression in diverse cancer populations. This study investigated HER2 expression in solid tumour samples and the feasibility of obtaining these results.

**Methods:**

Prospective consent was obtained at a Canadian tertiary academic cancer center from adult oncology patients who were referred for molecular genetic testing of malignant tissue samples. Standard HER2-targeted malignancies were considered breast and gastroesophageal, and were excluded from this study. Between July 2020 and November 2023, 499 samples of solid tumors underwent immunohistochemistry (IHC) HER2 staining. A median turnaround time (TAT) of 14 days would be considered feasible for clinical decision making.

**Results:**

The mean age (± SD) of participants was 67 ± 12.5 years, with 270 (54%) male and 229 (46%) female. HER2 protein expression was measured in 42 unique cancer types. IHC levels of 0, 1+, 2+, and 3+ were reported and were 43%, 12%, 35%, and 10% of all analyzable samples respectively (tissue inadequate in 3% of samples). The median TAT for HER2 expression results from time of request to result in release was 18 (interquartile range, 11 to 30) days.

**Conclusions:**

HER2 protein expression varies widely between different cancer types. TAT for HER2 IHC results was a median of 18 days, which is close to our feasibility cut-off.

## Introduction

Human epidermal growth factor receptor-2 (*HER2* or *ErbB2* or *cErBB2*) gene is a proto-oncogene that was identified in the 1980s [1, 2]. Soon after, several independent studies found that an EGFR-related gene is amplified in a human breast cancer cell line, and named it *HER2* [3]. Akiyama et al. [4] showed that the protein product of *HER2* gene has a tyrosine kinase activity. The HER2 protein belongs to a family of four main members (HER1, HER2, HER3, and HER4) which control cell growth, survival, and differentiation. The binding of ligand to the transmembrane HER2 receptor results in receptor dimerization, and autophosphorylation of tyrosine residues present within the cytoplasmic domain ultimately leading to initiation of downstream signaling pathways and resulting in cell proliferation and/or tumorigenesis.

Slamon et al. [5] found that the *HER2* gene is amplified in up to 30% of breast cancers and this correlated with poor survival, establishing it as a prognostic biomarker. HER2 receptor overexpression, either through gene amplification or through transcriptional deregulation, plays a pivotal role in transformation and tumorigenesis [6]. This made HER2 a predictive biomarker and a promising target for cancer treatment. In 1998, Herceptin was the first monoclonal antibody approved for HER2 positive metastatic breast cancer. Since then, the therapeutic armamentarium targeting HER2 has broadened to include new drugs, including tyrosine kinase inhibitors (TKIs), monoclonal antibodies and antibody-drug conjugates (ADCs). The current indications for drugs targeting HER2 also include HER2 “low” breast cancer, as well as, HER2 positive metastatic gastric/esophageal, colon and lung cancer [7–10].

The tests, to find if a patient with breast cancer will benefit from HER2 directed therapy, focus on identifying HER2 protein overexpression or gene amplification [copy number variation (CNV)], by immunohistochemistry (IHC) or in situ hybridization (ISH) respectively, as per ASCO-College of American Pathologists (CAP) guidelines [11]. Given the differences between HER2 expression in breast and gastric cancer, a different HER2 scoring system was developed and validated for gastric adenocarcinoma by Hofmann et al. [12] for the TOGA trial [13].

More studies are exploring HER2 targeted therapies in other solid cancers such as biliary tract, bladder, cervical, endometrial, ovarian, and pancreatic cancers [14–19]. As the indications of HER2 targeted treatment increase, an accurate assessment of HER2 status is critical in the face of heterogeneity of HER2 overexpression among histologies, as well as, intratumoral heterogeneity [20, 21]. Besides the biological heterogeneity, there are technical challenges such as standardization/validation of IHC and ISH techniques across histologies, lack of guidelines regarding interpretation of HER2 IHC in histologies other than breast and gastric cancer, and an increase in turnaround time (TAT) with increasing case burden on technicians and pathologists. These challenges warrant further characterization of HER2 expression in diverse cancer populations. Furthermore, with ADCs there is evidence of efficacy for patients not classically considered to be HER2 “positive”, stressing the importance of better understanding the spectrum of HER2 expression in all cancers [7]. This study investigated HER2 overexpression in solid tumour samples and the feasibility of obtaining these results at a tertiary care cancer centre.

## Materials and methods

### Patients

We included all patients with solid malignancies. Standard HER2-targeted malignancies were considered breast and gastric/esophageal adenocarcinoma, and were excluded from this study. Prospective consent was obtained from adult oncology patients referred to the London Regional Cancer Program at London Health Sciences Centre for IHC and molecular genetic testing of malignant tissue samples as part of the Precision Oncology at WesteRn University (POWER) study. The study was conducted in accordance with the Declaration of Helsinki and Good Clinical Practice. The study was approved by the institutional research ethics board (REB#108938).

### Assays

Formalin-fixed paraffin-embedded (FFPE) specimens were used. The histopathology of each specimen was confirmed on a freshly cut hematoxylin and eosin-stained slide by a board-certified pathologist. Tissues were micro-/macro-dissected when < 20% tumor cells were present (to enrich the sample for tumor). HER2 status was evaluated by IHC testing. For IHC positivity, analysis of samples was performed using the HercepTestTM kit (Dako; no. K520411-2, manual sample processing). All assays were performed on 4-µm tissue sections on positively charged slides. The samples were analyzed using Ruschoff/Hofmann criteria for gastroesophageal adenocarcinoma for HER2 positivity status, as established by the CAP guidelines [22].

### Statistical analysis

Data including patient gender, age, tumor type, source of tissue, and TAT was curated retrospectively from review of the charts and was summarized by descriptive analysis. TAT was calculated as the time of request for HER2 IHC to result release. It was assumed that a median TAT of ≤ 14 days would be considered feasible for clinical decision making.

## Results

### Patient and tumor characteristic

The mean age (± standard deviation, SD) of participants was 67 ± 12.5 years, with 270 (54%) males and 229 (46%) females (Table 1).

**Table 1:** HER2 immunohistochemistry results in diverse cancers

**Variable**	***N* (%) of patients**
Age, mean ± SD	67 ± 12.5 years
Gender (*N* = 499)	
Male Female	270 (54%) 229 (46%)
Number of tumor types tested, *N*	42
Inadequate tissue retrieved	15 (3%)
Source of tissue (*N* = 499)	
Biopsy Surgical resection	329 (66%) 170 (34%)
IHC levels (*N* = 484)	
0 1+ 2+ 3+	209 (43%) 56 (12%) 169 (35%) 50 (10%)
TAT for HER2 expression results (*N* = 489)	18 (IQR, 11 to 30) days

TAT: turnaround time; SD: standard deviation; IQR: interquartile range

Between July 2020 and November 2023, 499 samples of diverse solid tumors underwent IHC HER2 staining. Fifteen (3%) samples had inadequate archival tissue to complete the analysis. HER2 protein expression was measured in 42 unique cancer types. There was wide variation in sample size of each cancer type, ranging from 219 for pancreatic cancer samples to only one sample for thirteen cancers. The other common cancer types in this analysis were colorectal (*n* = 67), non-small cell lung cancer (NSCLC; *n* = 34), ovarian (*n* = 27), cholangiocarcinoma (*n* = 27), cervical (*n* = 12), mesothelioma (*n* = 10), prostate (*n* = 9), endometrial (*n* = 9), thyroid (*n* = 8), bladder (*n* = 7) and urothelial (*n* = 6) (Table S1).

### IHC score by cancer type

**Figure 1:**
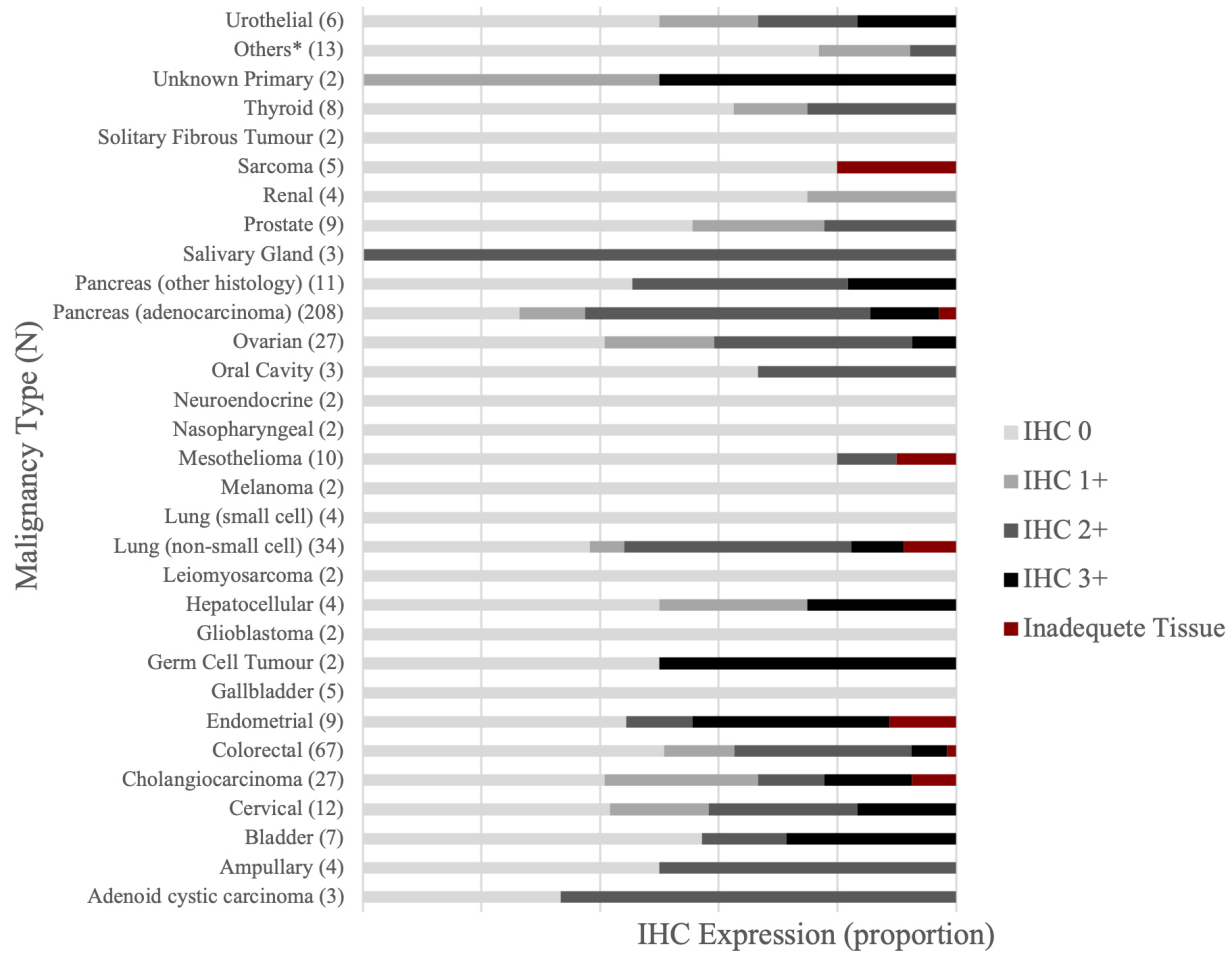
HER2 expression level distribution across diverse cancer types. ^*^: 1 sample each for adrenocortical, anal, anaplastic thymoma, chordoma, clear cell mandible, hypopharynx, meningioma, oropharyngeal, penile, primary peritoneal (mucinous), primary carcinoma, small bowel, and vulvar cancer. HER2: human epidermal growth factor receptor-2; IHC: immunohistochemistry

**Figure 2:**
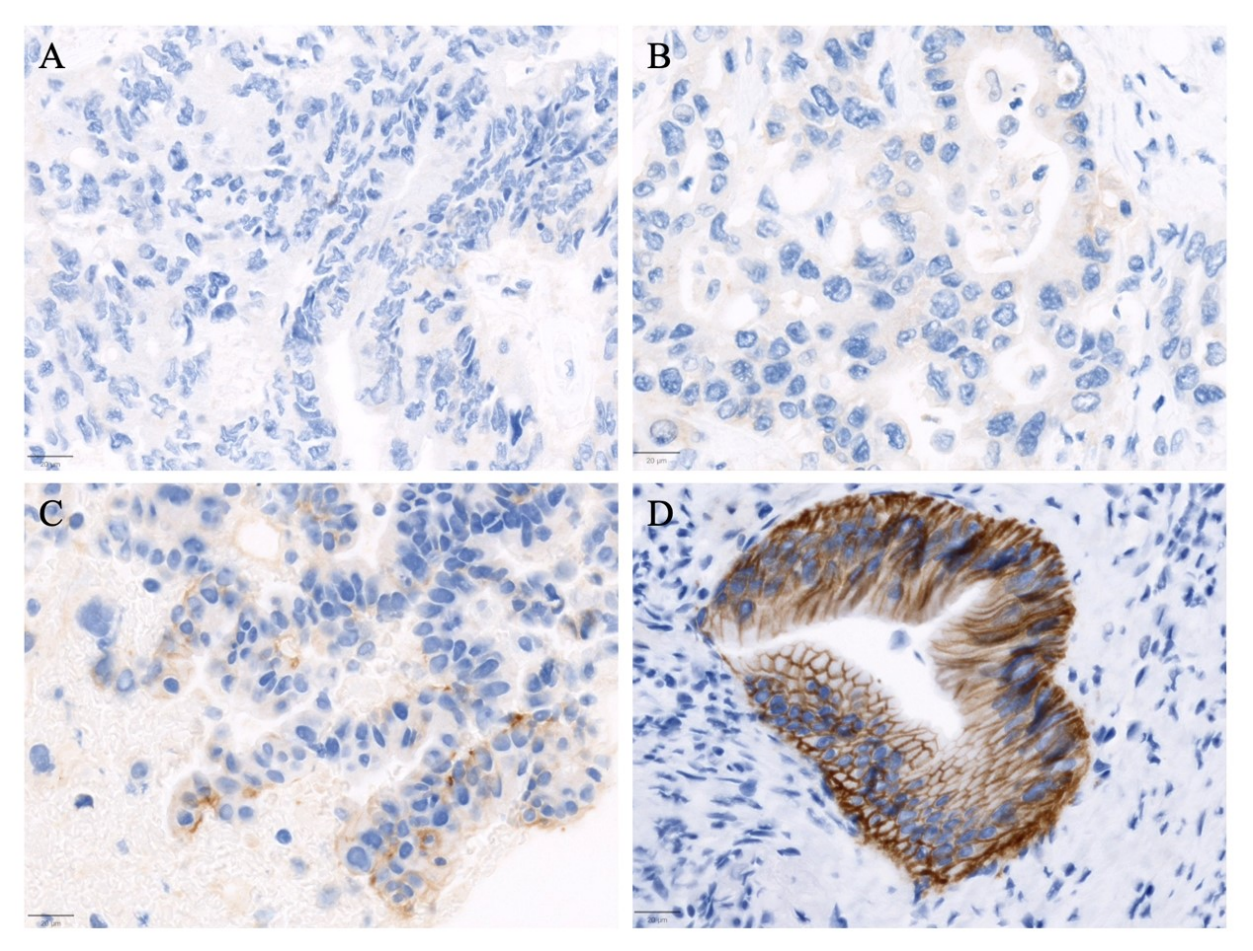
Representative immunohistochemistry staining of HER2 (A0485) categorized into four scoring levels. (A) Case of metastatic colorectal adenocarcinoma with no HER2 expression (scored as 0); (B) exhibits a case of a pancreaticobiliary type adenocarcinoma of the ampulla of Vater with weak and incomplete membranous staining in more than 10% of tumor cells, scored as 1+; (C) pancreatic adenocarcinoma with moderate complete, basolateral, or lateral membranous staining in more than 10% of tumor cells, classified as 2+; (D) a case of duodenal adenocarcinoma with strong complete, basolateral, or lateral membranous staining in more than 10% of tumor cells, scored as 3+. The images were captured at an objective magnification of 20X (scale bar = 20 μm)

The IHC scores in all samples respectively were 0 (43%), 1+ (12%), 2+ (35%), and 3+ (10%). The IHC score varied widely with cancer type (Figure 1, Figure 2, and Table S1). Among the cancer types for which 7 or more samples were tested, an IHC 3+ score was demonstrated in endometrial (33.3%), bladder (28.6%), cervical (16.7%), cholangiocarcinoma (14.8%), pancreas (11.9%), NSCLC (8.8%), ovarian (7.4%), colorectal (6.0%), mesothelioma (0%), thyroid (0%), and prostate (0%). There were five samples for gallbladder cancer and the IHC score was 0 in all. Two samples from cancers of unknown primary showed IHC score of 1+ and 3+ each.

### Turn-around time

The median TAT for HER2 IHC expression results was 18 (interquartile range, 11 to 30) days. The source of the tissue was a biopsy in 329 (66.3%) cases or a surgical specimen in 170 (34.7%) cases. There was no significant difference in the TAT based on source of tissue, i.e., biopsy versus surgical resection (*P* = 0.7013). The majority of samples were collected from London Health Sciences Center (447, 89.6%) and there was no significant difference in time for results compared to samples collected outside hospitals (*P* = 0.0939).

## Discussion

The oncogenic potential of HER2 amplification/overexpression extends to cancers other than breast and esophageal/gastric adenocarcinoma. Historically, the prognosis of HER2 driven cancers has been bleak (pre-trastuzumab era). The clinical outcomes are better in patients who receive matched targeted treatment compared to those who don’t [23]. Recently, new indications for HER2 directed treatment have been established in NSCLC and colon cancers, and an expanded indication in HER2 low breast cancer with ongoing studies to exploit the HER2 pathway in other cancers [18]. Novel therapeutics targeting HER2, like ADCs, TKIs (e.g., tucatinib) and bispecific antibodies (e.g., zanidatamab) are promising and are likely to change treatment landscape in this patient population [18, 19, 24, 25]. Hence, there is an unmet need of standardizing the methodology for HER2 positivity in diverse cancers as the overexpression differs widely across cancer types. In this study, we looked at feasibility of HER2 IHC expression across various cancers, including TAT.

We tested 499 samples with a wide variation in sample size of each cancer type, including, pancreatic, colorectal, NSCLC, ovarian, cholangiocarcinoma, endometrial, thyroid, urothelial, prostate, and bladder cancer. The IHC was positive (3+) in 10% of all samples; 33.3%, 28.6%, 16.7% and 14.8% for endometrial, bladder, cervical cancer, and cholangiocarcinoma respectively.

The variability in rates of HER2 IHC expression in particular cancer types between our study and other studies can be explained by the lack of standardized and validated IHC score in these cancers (except breast and gastric), as well as, geographic and ethnic factors [26, 27]. Other studies have looked at HER2 overexpression in diverse cancers. Yan et al. [28] examined 37,992 samples for HER2 by IHC (ISH done for 21,642). Of these 18,262 had tumors other than breast/gastric cancer. HER2 overexpression was seen in 2.7% of samples. The HER2 positivity rates for bladder carcinomas, gallbladder, extrahepatic cholangiocarcinoma, cervical, uterine, and testicular cancers was 12.4%, 9.8%, 6.3%, 3.9%, 3.0%, and 2.4%, respectively [28]. Kujtan et al. [29] evaluated HER2 activity measured by IHC, mRNA expression and CNV across various solid tumors (*n* = 856). 43% patients had positive by IHC (3+), 30% had high *HER2* mRNA expression and 5% had amplification by CNV. The percentage of HER2 IHC 3+ or 2+/CNV positive was 18.5%, 8.1%, 6.7%, 10%, 16%, 28.6%, 5.1%, and 3.1% in breast cancer, NSCLC, colorectal, esophago-gastric, urothelial/bladder, biliary, ovarian cancer and pancreas [29].

With any targeted treatment, TAT for tests such as HER2 IHC, is extremely important to be able to start the targeted treatment. Delay of the HER2 targeted treatment may have a negative impact on clinical outcomes [30]. The TAT for HER2 results should be short to prevent any delay in the start of treatment, especially in malignancies in which HER2 targeted treatment is indicated as first line. The ASCO-CAP guidelines recommends that laboratories must provide clinically appropriate TAT, and suggests a benchmark of 90% of reports available within 10 working days from the date of procedure or specimen acquisition [22]. The median TAT at our centre, in this study, is 18 calendar days which is slightly beyond the suggested benchmark for clinical decision making. This could be explained by the fact that non-interventional research pathology requests at our institution are treated with a lower priority, and it is reasonable to expect if this testing were part of standard pathways the benchmark would be met.

Recent trials, such as DESTINY-PanTumor02, have tested the efficacy and safety of trastuzumab deruxtecan (T-DXd) in patients with HER2-expressing solid tumors. Although, the patients with IHC 3+ had maximum benefit in terms of objective response rate (ORR), patients with IHC 2+ also had some benefit, without requiring ISH positivity as is done for the classical cut-off for benefit with trastuzumab in breast and gastric cancers [18]. Also, IHC scoring system on a biopsy, for gastric cancer, only requires a cluster of at least 5 cohesive cells with membrane reactivity to be positive (further categorization as 1+, 2+, or 3+ based on intensity and pattern of staining). This highlights the fact that the eligibility criteria for HER2 targeted treatment depends on the specific cancer type, drug, and its mechanism of action. Hence, the scoring criteria needs validation in each cancer type and for each individual drug. Although transcriptional patterns and multi-omics data may help in identifying potential candidates for treatment, in the absence of resources, IHC remains a pragmatic and validated test to be used for determining who is most likely to benefit from HER2 targeted therapy.

Additional trials have examined zanidatamab across HER2-expressing tumors and in biliary tract cancers. In all tumour types the clinical benefit rate was above 50% and the phase I study included patients with all levels of HER2-expression and allowed previous HER2 targeted therapy [31]. In a biliary tract specific study, patients with HER2-expression of IHC 2+ and 3+ had an ORR of 41% [19]. Pertuzumab + trastuzumab has demonstrated efficacy in numerous tumor types through the MyPathway and TAPUR studies, including an ORR and disease control rate (DCR) of 23% and 51% respectively in biliary tract cancers. The ORR and DCT was 32% and 44%, respectively, in colorectal cancer [32–34]. Tucatinib with trastuzumab has also demonstrated activity across tumor types with an ORR of 39% and DCR of 71% in selected patients with HER2-expressing refractory colon cancer; ORR of 47% and DCR of 77% in patients with previously treated HER2-expressing biliary tract cancers [9, 35]. These studies emphasize the efficacy of HER2 targeting across tumor types with varying levels of HER2-expression.

There are several limitations to our single centre study. The sample size of some tumour types was quite limited with only one sample for thirteen cancers. There was also wide variation in sample size of each cancer type. Secondly, ISH was not done for IHC 2+ samples because of resource limitations, which is needed for determining positivity by classical cut-offs. HER2 mutation status is also not available for these patients, although *HER2* mutation and *HER2* amplification are distinct oncogenic drivers. The overlap of *HER2* mutation and *HER2* amplification is rare and they are distinct therapeutic targets [36]. There may be a selection bias of the specimens. Lastly, the study does not report if the HER2 IHC positivity changed the clinical management, such as a HER2 directed treatment, although the center does have an open trial for patients with HER2 aberrations [37].

### Conclusions

In this study, we tested HER2 protein expression in diverse cancers. We found that HER2 protein expression, as measured by IHC, varies widely between different cancer types. TAT for HER2 IHC results was a median of 18 days, which is close to feasibility cut off and clinically appropriate for clinical decision making. This value appears unaffected by tissue sample source or originating hospital, however there was a large range in release time, which can have clinical implications when making time-sensitive treatment decisions. Further efforts should aim at consensus criteria for HER2 evaluation in various cancers.

## Abbreviations

ADCs: antibody-drug conjugates
CAP: College of American Pathologists
CNV: copy number variation
DCR: disease control rate
HER2: human epidermal growth factor receptor-2
IHC: immunohistochemistry
ISH: in situ hybridization
ORR: objective response rate
TAT: turnaround time

## Supplementary materials

The supplementary material for this article is available at: https://www.explorationpub.com/uploads/Article/file/1002265_sup_1.pdf.

## Declarations

### Availability of data and materials

Data are available on reasonable request.

### Copyright

© The Author(s) 2024.
