# Supplementary material for: Evaluation of HER2 immunohistochemistry expression in non-standard solid tumors from a Single-Institution Prospective Cohort
Source: Explor Target Antitumor Ther. 2024 Aug 22;5(5):1100–9. doi: 10.37349/etat.2024.00265 (PMC11438559; doi:10.37349/etat.2024.00265)
Supplement: Supplementary file 1 [file 1002265_sup_1.pdf]

Table S1. HER2 Immunohistochemistry Results in Diverse Cancers

**Malignancy Type (N)**	**HER2 Expression Level**				
	**0 N (%)**	**1+ N (%)**	**2+ N (%)**	**3+ N (%)**	**Inadequate Tissue N (%)**
Adenoid cystic carcinoma (3*)	33.3	0.0	66.7	0.0	0.0
Adrenocortical (1)	100.0	0.0	0.0	0.0	0.0
Ampullary (4)	50.0	0.0	50.0	0.0	0.0
Anal (1)	0.0	100.0	0.0	0.0	0.0
Anaplastic Thymoma (1)	100.0	0.0	0.0	0.0	0.0
Bladder (7)	57.1	0.0	14.3	28.6	0.0
Cervical (12)	41.7	16.7	25.0	16.7	0.0
Cholangiocarcinoma (27)	40.7	25.9	11.1	14.8	7.4
Chordoma (1)	100.0	0.0	0.0	0.0	0.0
Clear Cell Mandible (1)	0.0	100.0	0.0	0.0	0.0
Colorectal (67)	50.7	11.9	29.9	6.0	1.5
Endometrial (9)	44.4	0.0	11.1	33.3	11.1
Gallbladder (5)	100.0	0.0	0.0	0.0	0.0
Germ Cell Tumour (2)	50.0	0.0	0.0	50.0	0.0
Glioblastoma (2)	100.0	0.0	0.0	0.0	0.0
Hypopharynx (1)	100.0	0.0	0.0	0.0	0.0
Hepatocellular (4)	50.0	25.0	0.0	25.0	0.0
Leiomyosarcoma (2)	100.0	0.0	0.0	0.0	0.0
Lung (non-small cell) (34)	38.2	5.9	38.2	8.8	8.8
Lung (small cell) (4)	100.0	0.0	0.0	0.0	0.0
Melanoma (2)	100.0	0.0	0.0	0.0	0.0

Meningioma (1)	100.0	0.0	0.0	0.0	0.0
Mesothelioma (10)	80.0	0.0	10.0	0.0	10.0
Nasopharyngeal (2)	100.0	0.0	0.0	0.0	0.0
Neuroendocrine (2)	100.0	0.0	0.0	0.0	0.0
Oral Cavity (3)	66.7	0.0	33.3	0.0	0.0
Oropharyngeal (1)	0.0	0.0	100.0	0.0	0.0
Ovarian (27)	40.7	18.5	33.3	7.4	0.0
Pancreas (adenocarcinoma) (208)	26.4	11.1	48.1	11.5	2.9
Pancreas (other histology) (11)	45.5	0.0	36.4	18.2	0.0
Penile (1)	100.0	0.0	0.0	0.0	0.0
Primary peritoneal (mucinous) (1)	100.0	0.0	0.0	0.0	0.0
Pituitary carcinoma (1)	100.0	0.0	0.0	0.0	0.0
Prostate (9)	55.6	22.2	22.2	0.0	0.0
Renal (4[#])	75.0	25.0	0.0	0.0	0.0
Sarcoma (5)	80.0	0.0	0.0	0.0	20.0
Salivary Gland (3[$])	0.0	0.0	100.0	0.0	0.0
Small Bowel (1)	100.0	0.0	0.0	0.0	0.0
Solitary fibrous tumor (2)	100.0	0.0	0.0	0.0	0.0
Thyroid (8)	62.5	12.5	25.0	0.0	0.0
Unknown Primary (2)	0.0	50.0	0.0	50.0	0.0
Vulva (1)	100.0	0.0	0.0	0.0	0.0
Urothelial (6)	50.0	16.7	16.7	16.7	0.0

N: number of cases; %: Percentage of cases; HER2: human epidermal growth factor receptor 2. [*]primary in trachea, hard palate and larynx (one each); #clear cell (n-2), chromophobe (n-1) and unclassified (n-1); $polymorphous adenocarcinoma (n-1), acinic cell carcinoma (n-1) and undifferentiated carcinoma of parotid (n-1)
